# Supplementary figures and images for: Optimizing the in vitro production of immunomodulatory cells for the induction of tolerance in solid organ transplantation
Source: PLoS One. 2025 Nov 7;20(11):e0333356. doi: 10.1371/journal.pone.0333356 (PMC12594342; doi:10.1371/journal.pone.0333356)

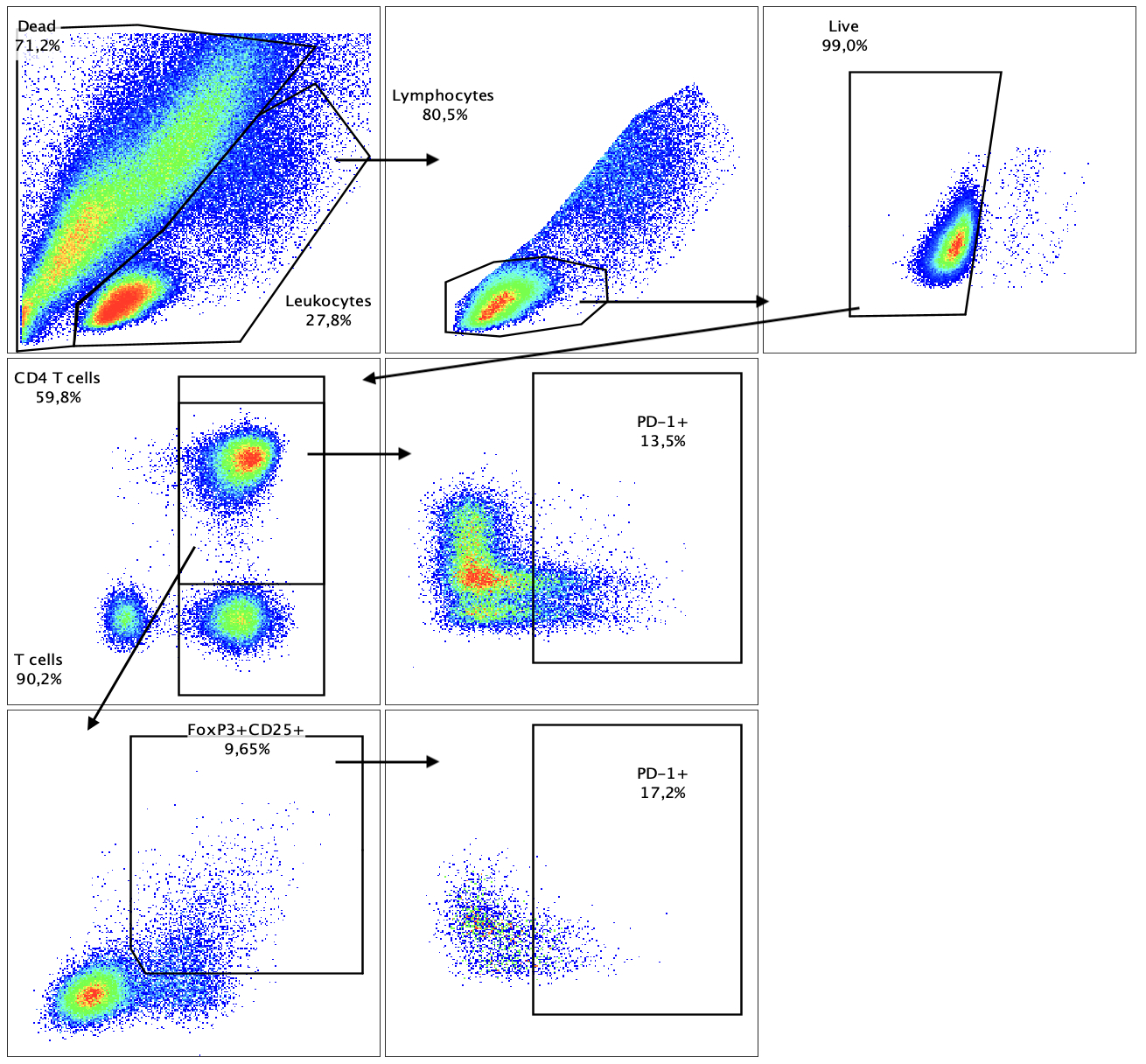

Supplement: S1 Fig — CD4 + T cell and Treg gating with activation marker PD-1. (TIF) [file pone.0333356.s001.tif]

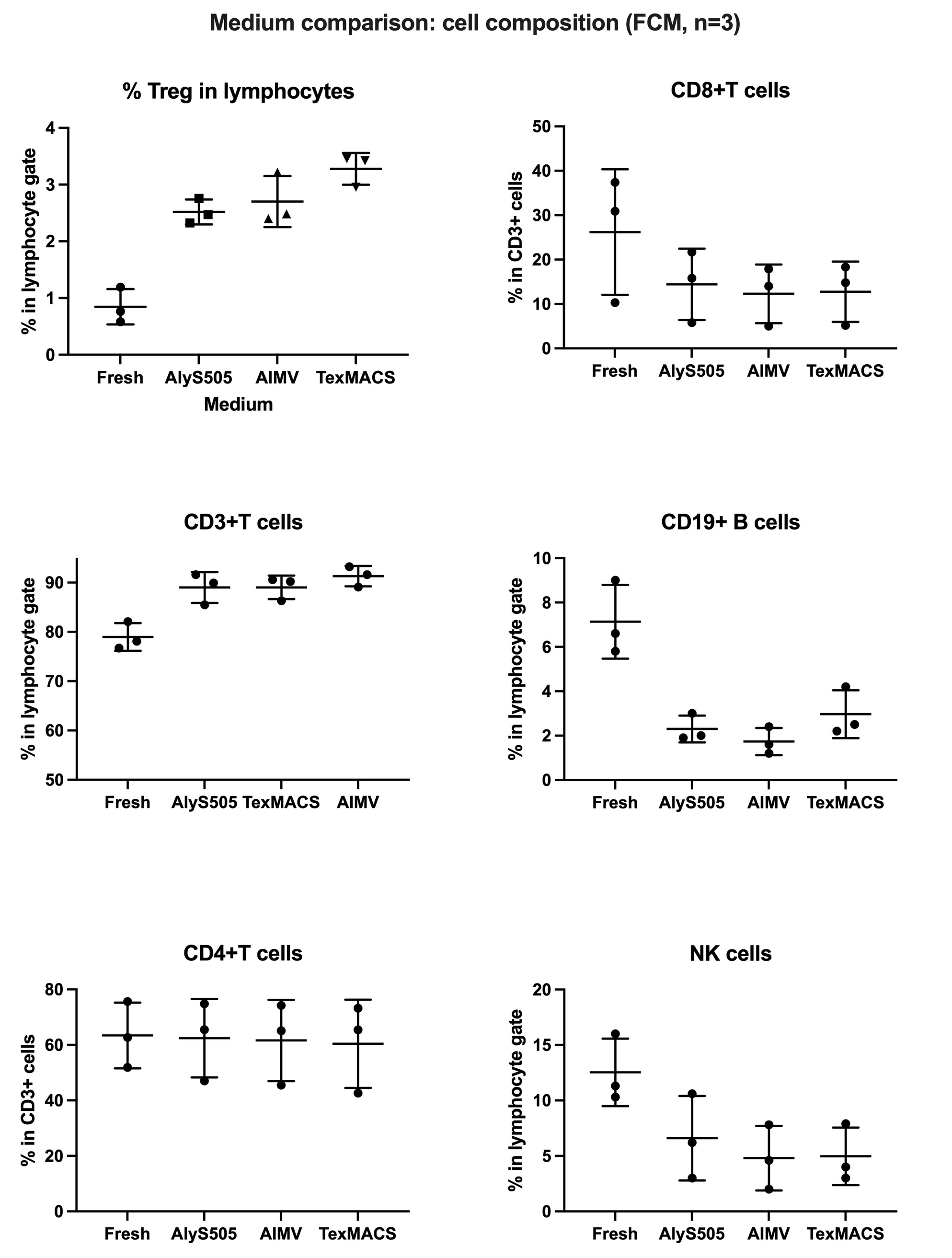

Supplement: S2 Fig — Comparison of DSIMC generation in three different media (AlyS505, AIM-V, TexMACS) compared to freshly separated PBMC. CD3 + , CD19 + , NK cells and Treg percentage of total lymphocytes. CD4+ and CD8 + cells of CD3 + cells. No significant differences. FCM. (n = 3). (TIF) [file pone.0333356.s002.tif]

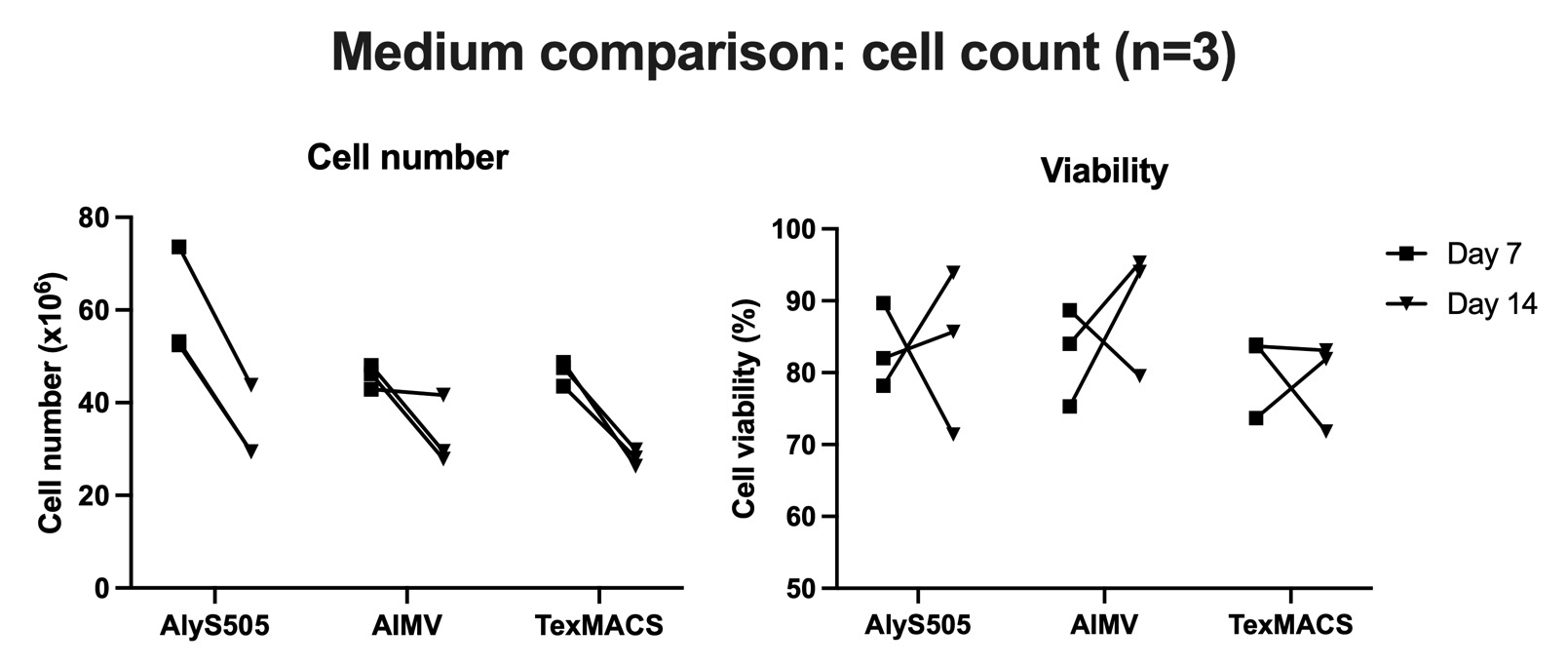

Supplement: S3 Fig — Comparison of DSIMC generation in three different media (AlyS505, AIM-V, TexMACS) over two weeks of culture. Cell numbers in million cells. Viability determined through live/dead staining (Trypan Blue) and presented as unstained live cells divided by stained dead cells. No significant differences. Manual cell counting. (n = 3). (TIF) [file pone.0333356.s003.tif]

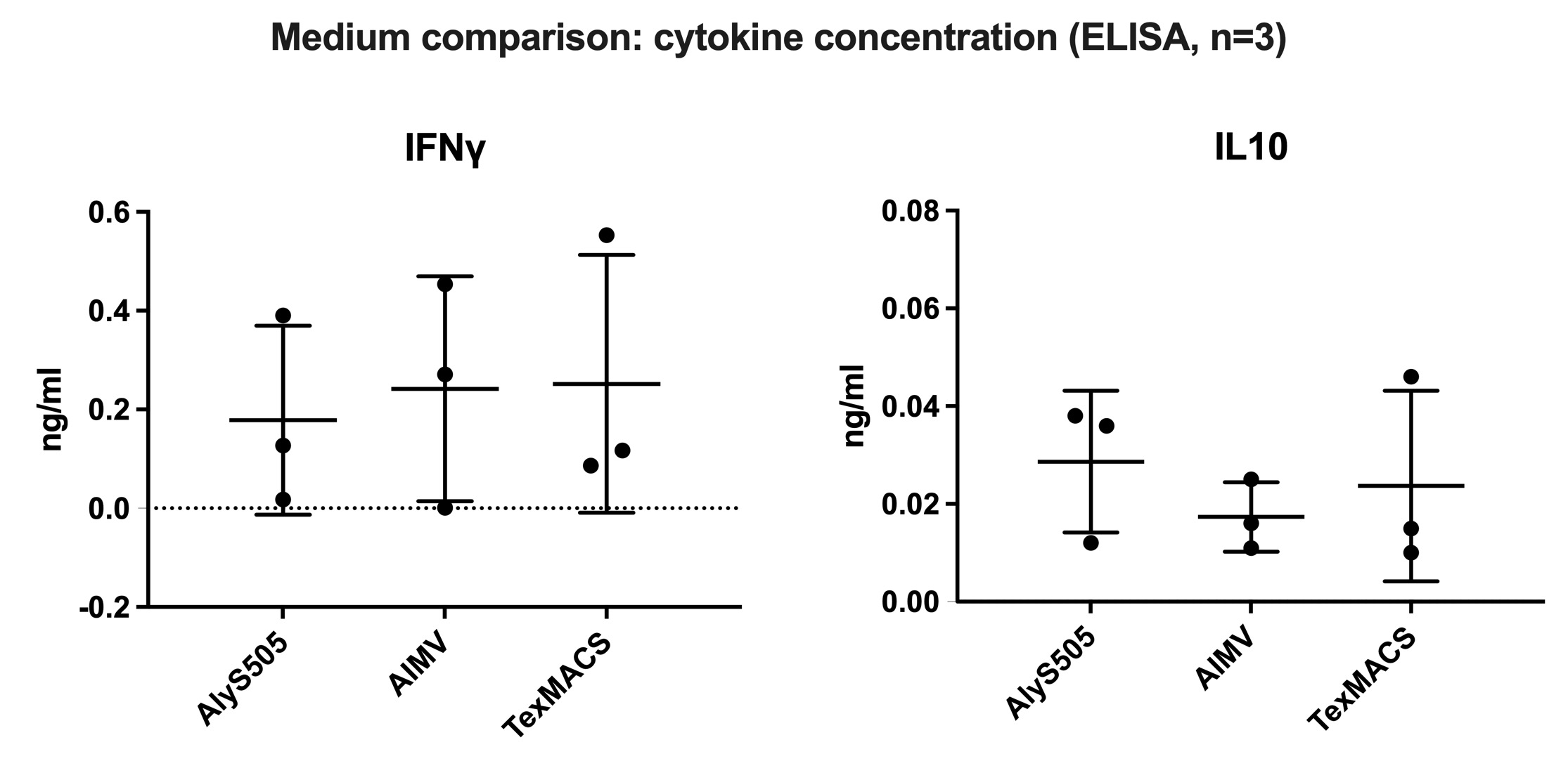

Supplement: S4 Fig — Comparison of DSIMC generation in three different media (AlyS505, AIM-V, TexMACS). IFNγ and IL-10 in ng/mL culture supernatant on day 14. No significant differences. ELISA. (n = 3). (TIF) [file pone.0333356.s004.tif]

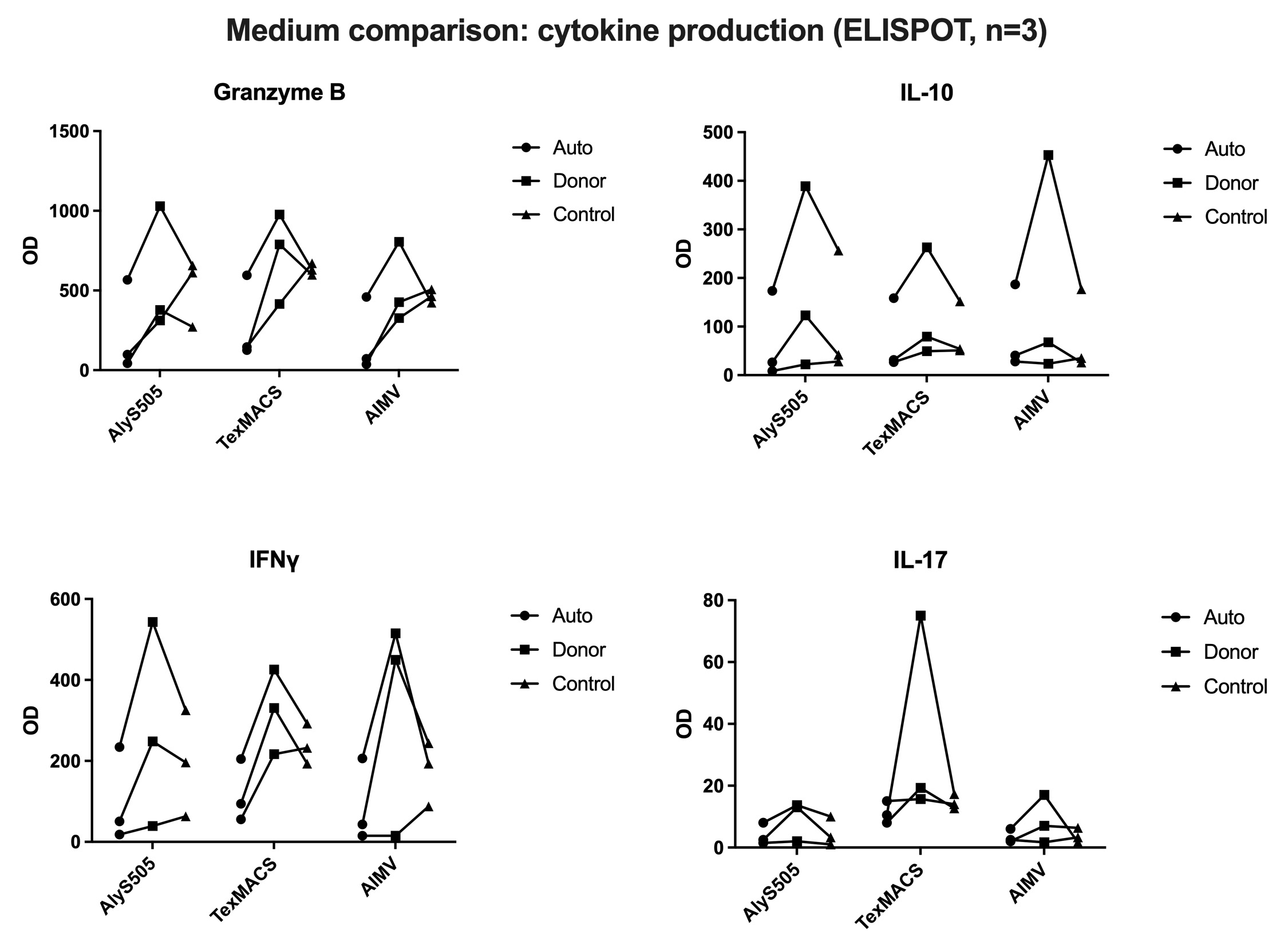

Supplement: S5 Fig — Comparison of DSIMC generation in three different media (AlyS505, AIM-V, TexMACS). Granzyme B, IFNγ, IL-17 and IL-10 production shown as total SFU (spot forming units) per million cells. Autologous cells added to DSIMC as negative control, donor stimulator cells added to DSIMC as specific proliferation activator, no cells added to controls. No significant differences. ELISPOT. (n = 3). (TIF) [file pone.0333356.s005.tif]

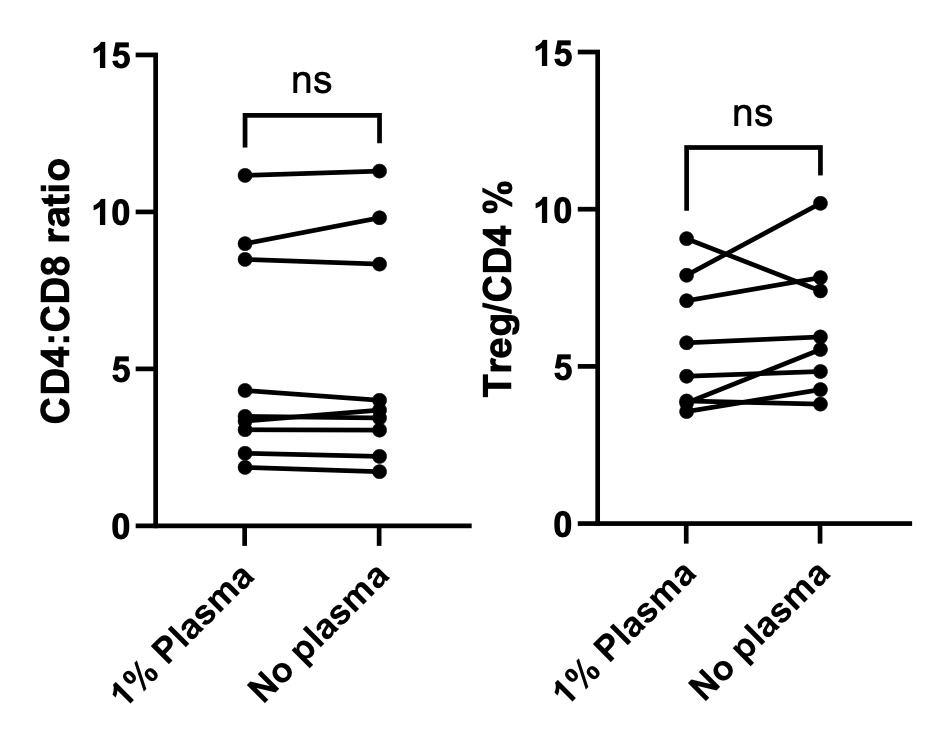

Supplement: S6 Fig — Comparison of CD4/CD8 ratio and Treg of CD4 + T cells in DSIMC generated with or without 1% autologous plasma added to the culture medium. No significant differences. FCM. (n = 9). (TIF) [file pone.0333356.s006.tif]

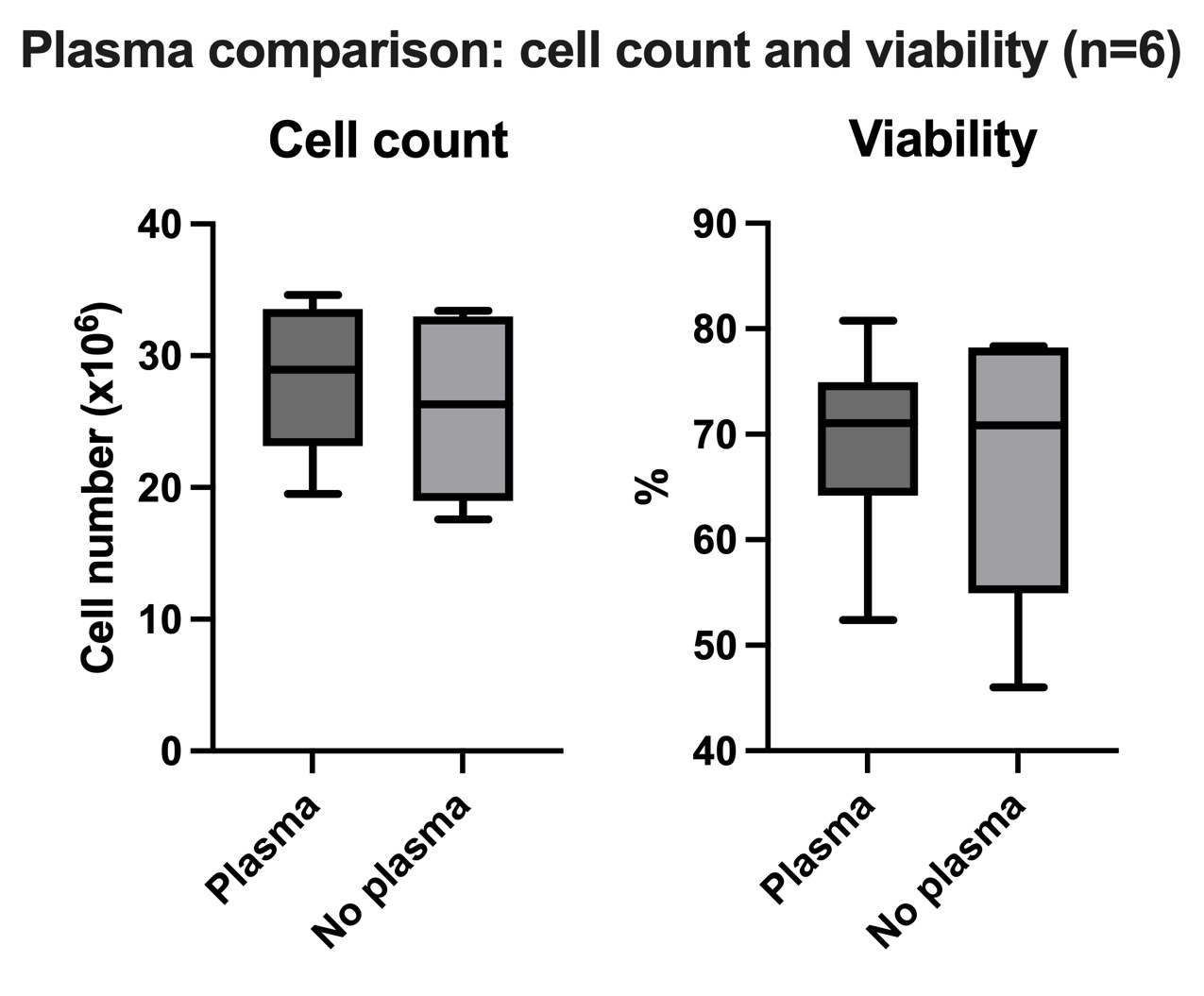

Supplement: S7 Fig — Comparison of DSIMC generation after two weeks of culture with or without 1% autologous plasma added to the culture medium. Cell numbers in million cells. No significant differences. Automated cell counter with nuclear staining and live/dead marker. (n = 6). (TIF) [file pone.0333356.s007.tif]

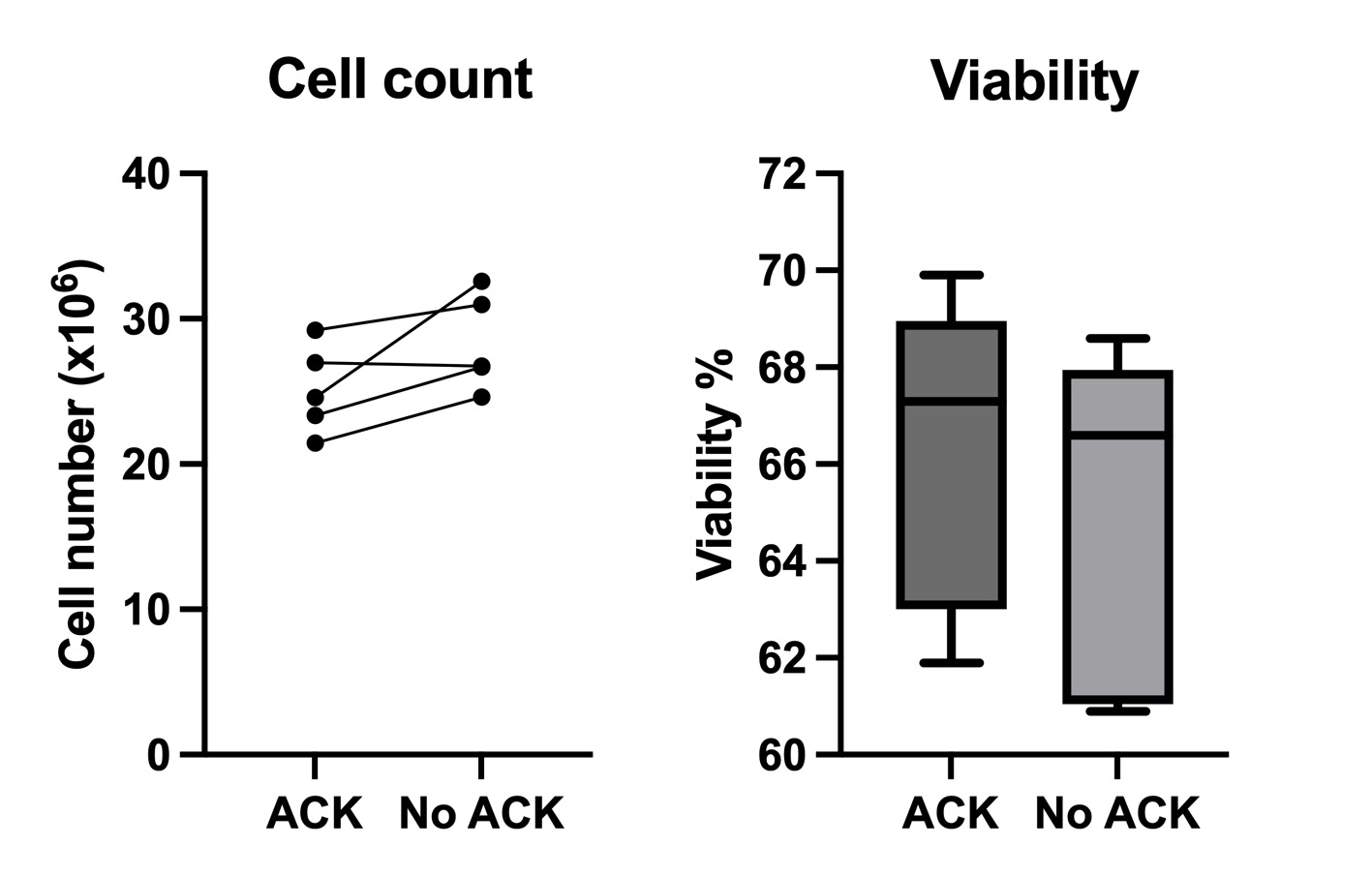

Supplement: S8 Fig — Comparison of DSIMC generation after two weeks of culture with or without ACK-treated PBMC. Cell numbers in million cells. No significant differences. Automated cell counter with nuclear staining and live/dead marker. (n = 5). (TIF) [file pone.0333356.s008.tif]

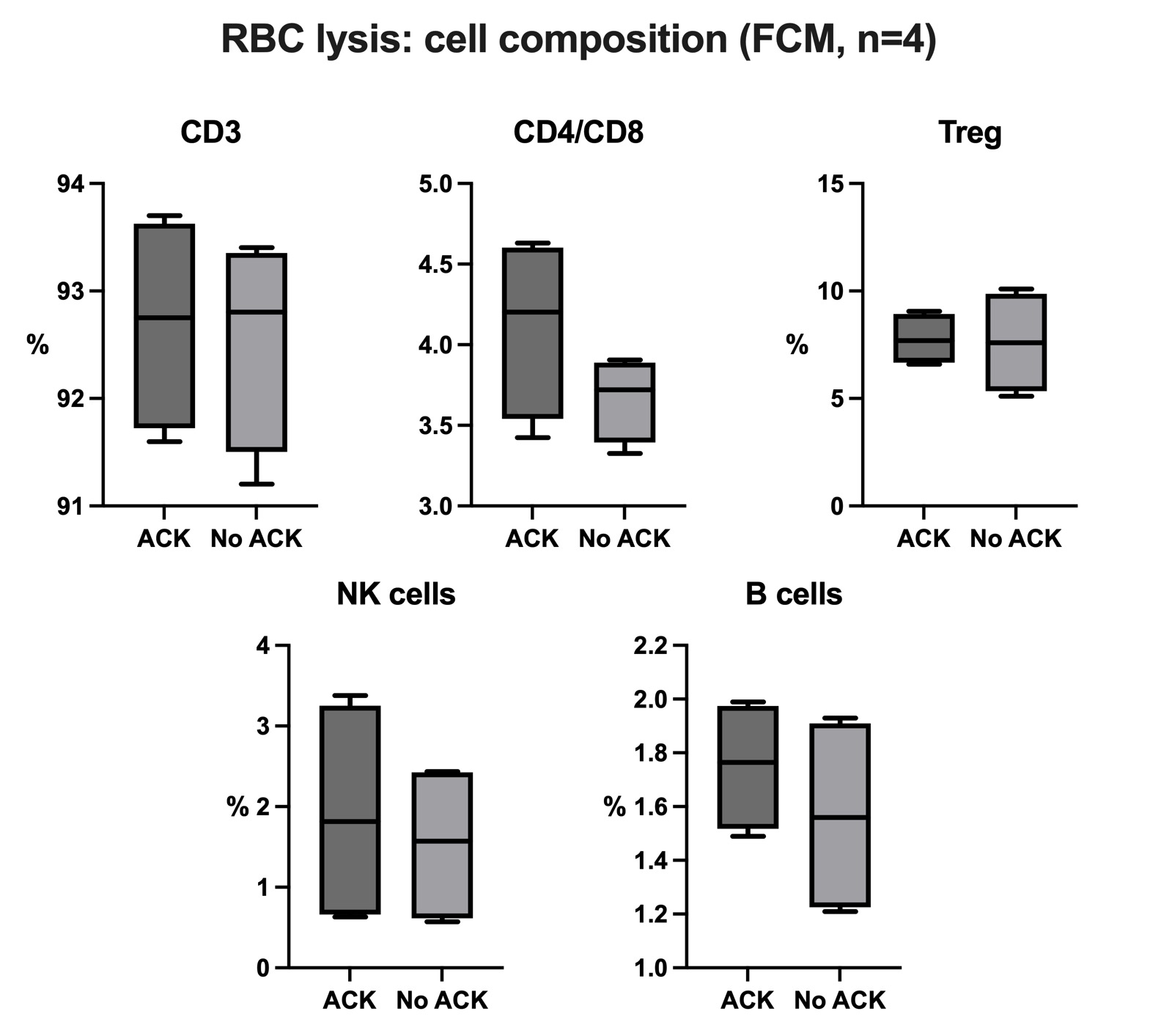

Supplement: S9 Fig — Comparison of DSIMC generation after two weeks of culture with or without ACK-treated PBMC. CD3 + T cells, regulatory T cells, NK cells, and B cells as percentage of total lymphocytes. No significant differences. FCM. (n = 4). (TIF) [file pone.0333356.s009.tif]

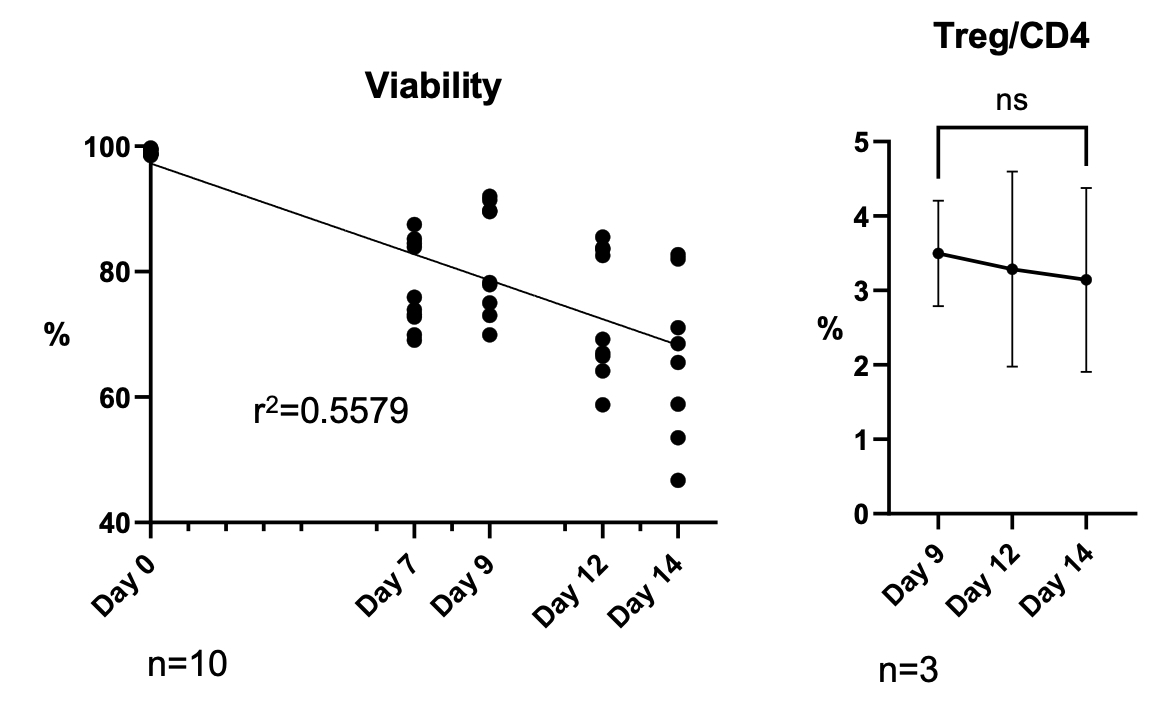

Supplement: S10 Fig — Comparison of viability and Treg percentage over time. (Left) Differences in viability stabilized over time. Automated cell counter with nuclear staining and live/dead marker, linear regression. (n = 10). (Right) No significant differences in percentage of Treg of total CD4 + T cells over time. FCM, paired t-test. (n = 3). (TIF) [file pone.0333356.s010.tif]

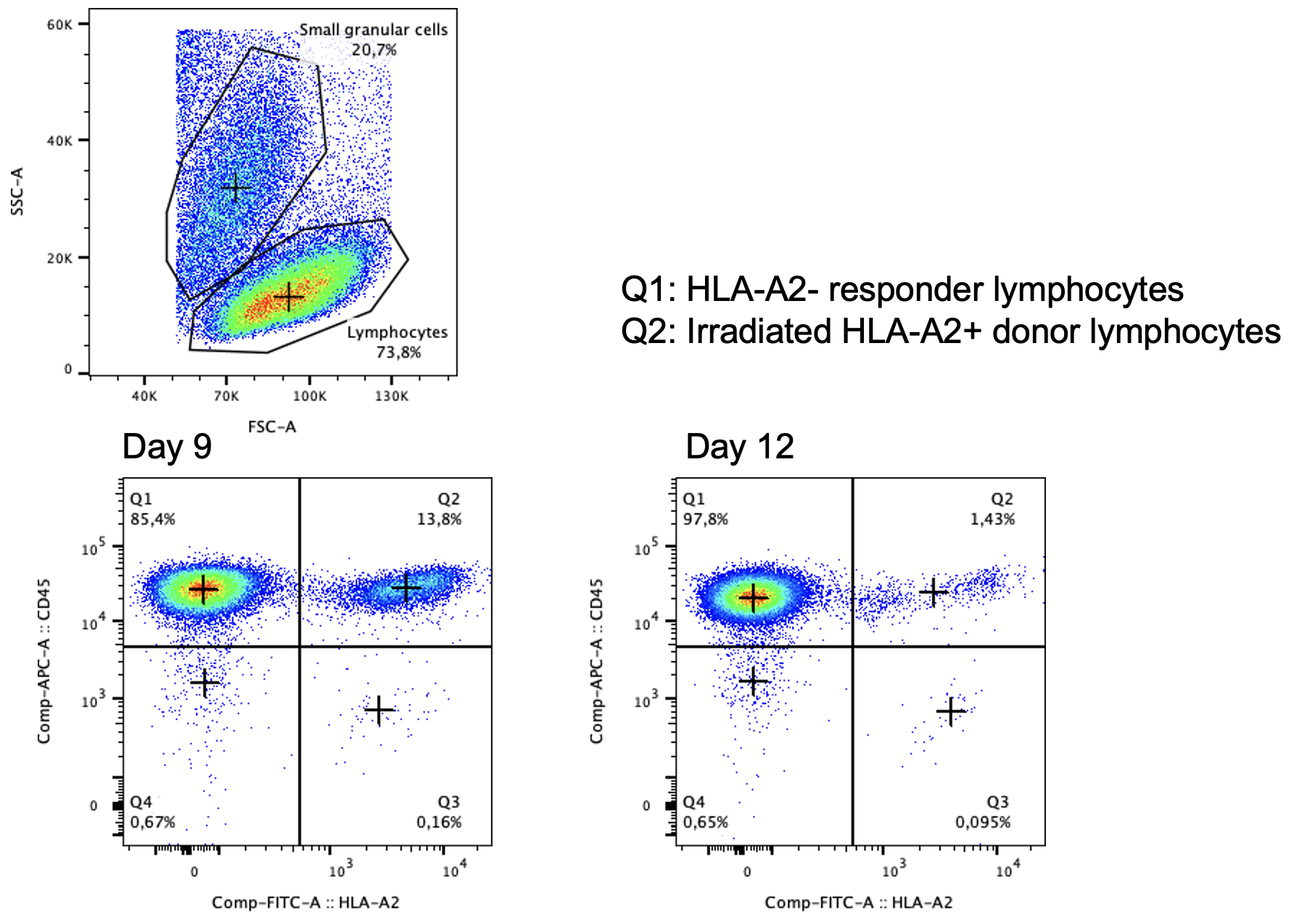

Supplement: S11 Fig — In this culture, irradiated HLA-A2 + stimulator cells reduce from 13.8% to 1.43% of the total T cell compartment. (TIF) [file pone.0333356.s011.tif]

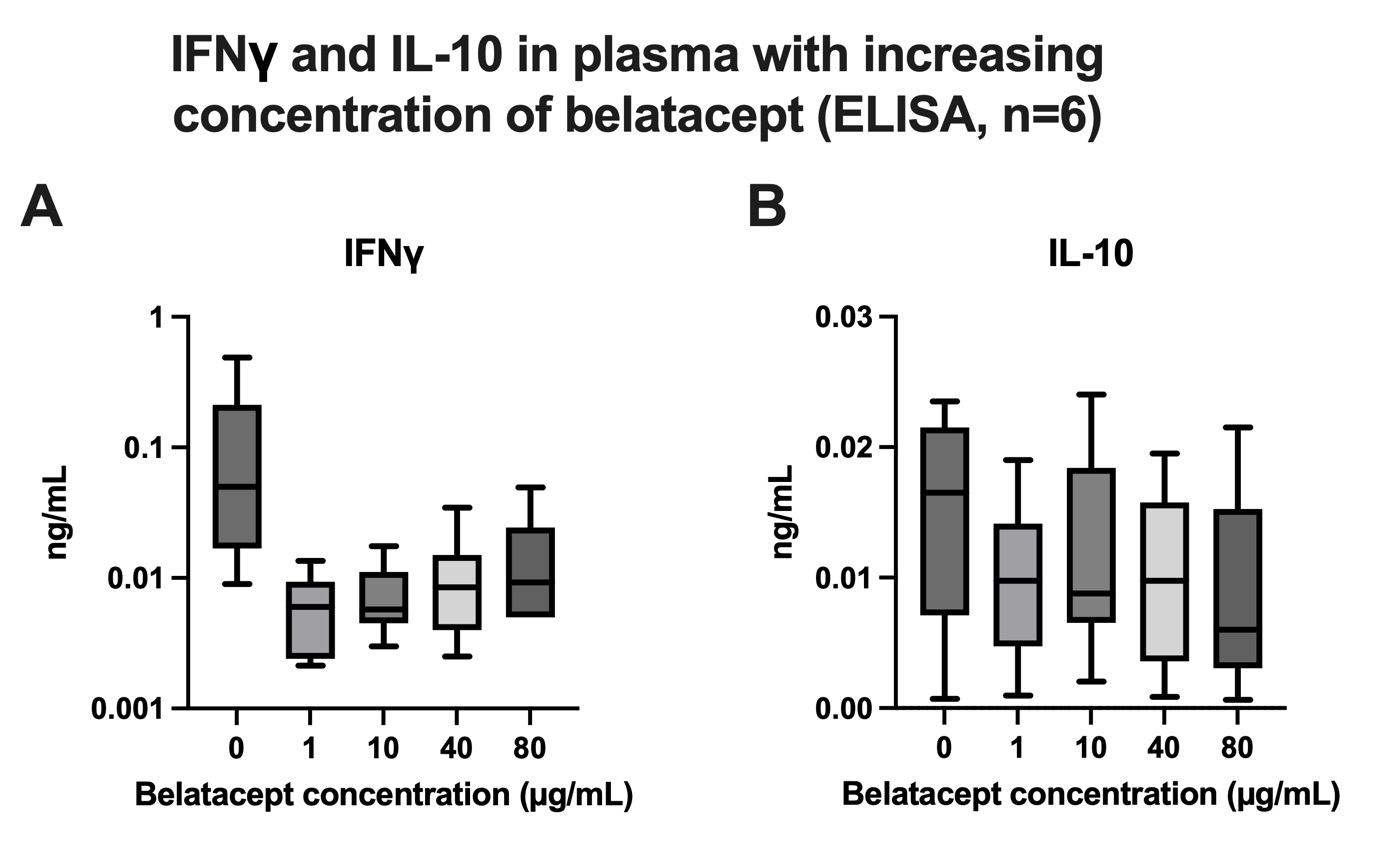

Supplement: S12 Fig — Effect of increasing concentration of belatacept in culture after two weeks on the concentration of cytokines in supernatant (ng/mL). No significant differences. Titration of belatacept as μg/million cells. ELISA. (n = 6). (TIF) [file pone.0333356.s012.tif]

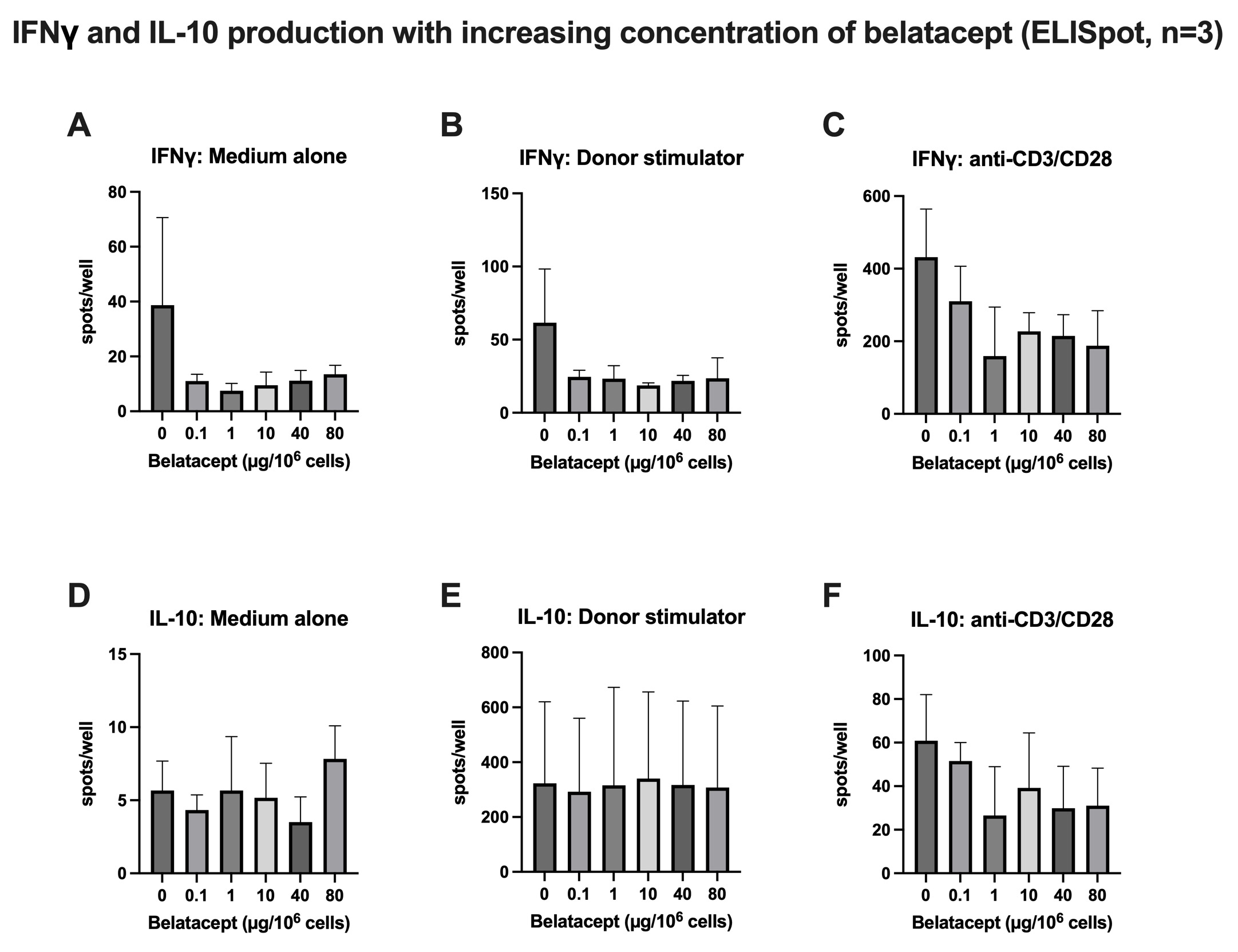

Supplement: S13 Fig — Comparison of DSIMC generation with increasing concentration of belatacept on production of cytokines in SFU (spot forming units). (A/D) without stimulation, (B/E) with specific stimulation, (C/F) with general stimulation. No significant differences. ELISpot. (n = 3). (TIF) [file pone.0333356.s013.tif]

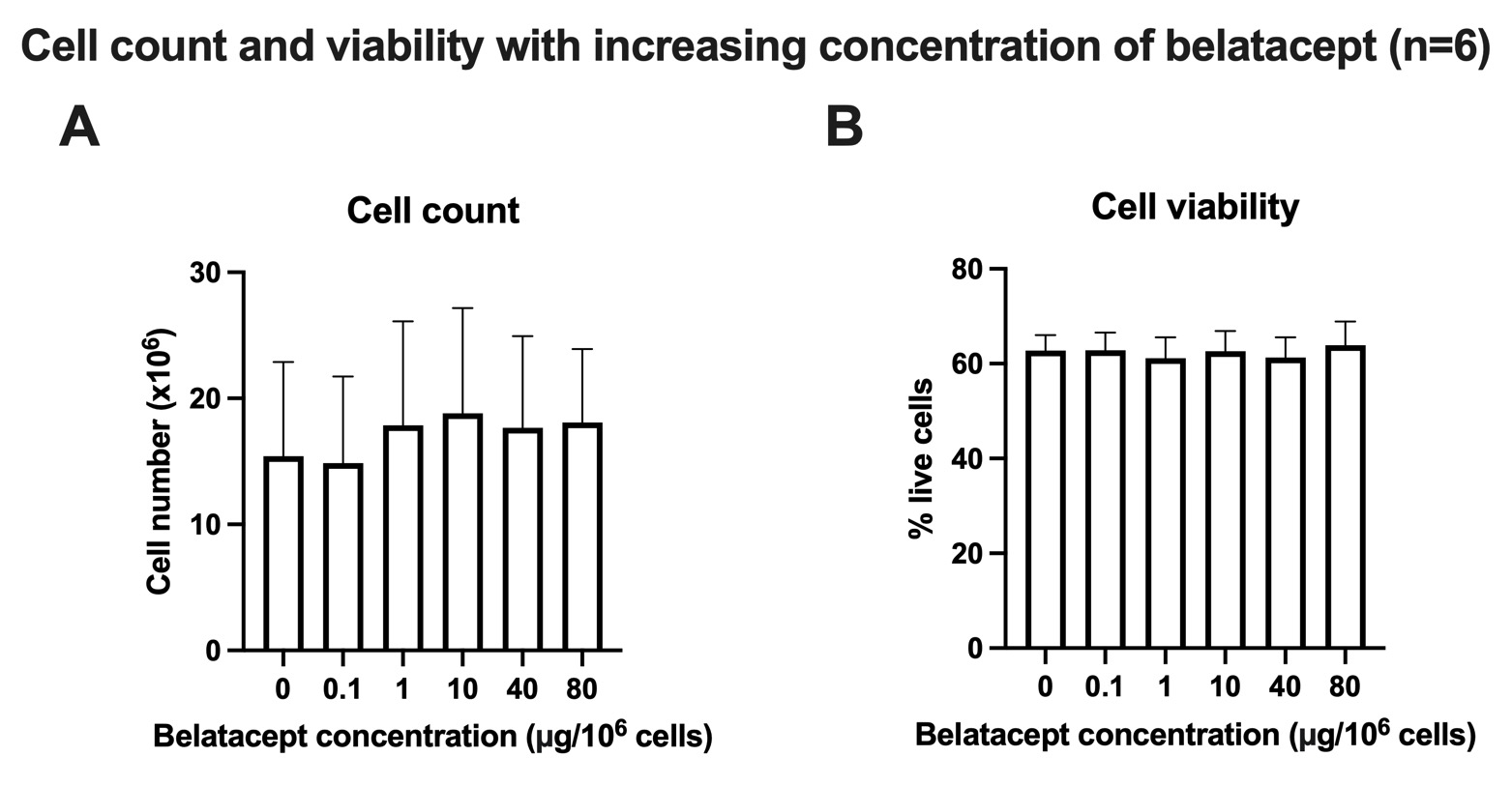

Supplement: S14 Fig — Comparison of DSIMC generation with increasing concentration of belatacept on cell numbers and viability after 14 days of culture. No significant differences. Automated cell counter with nuclear staining and live/dead marker. (n = 6). (TIF) [file pone.0333356.s014.tif]

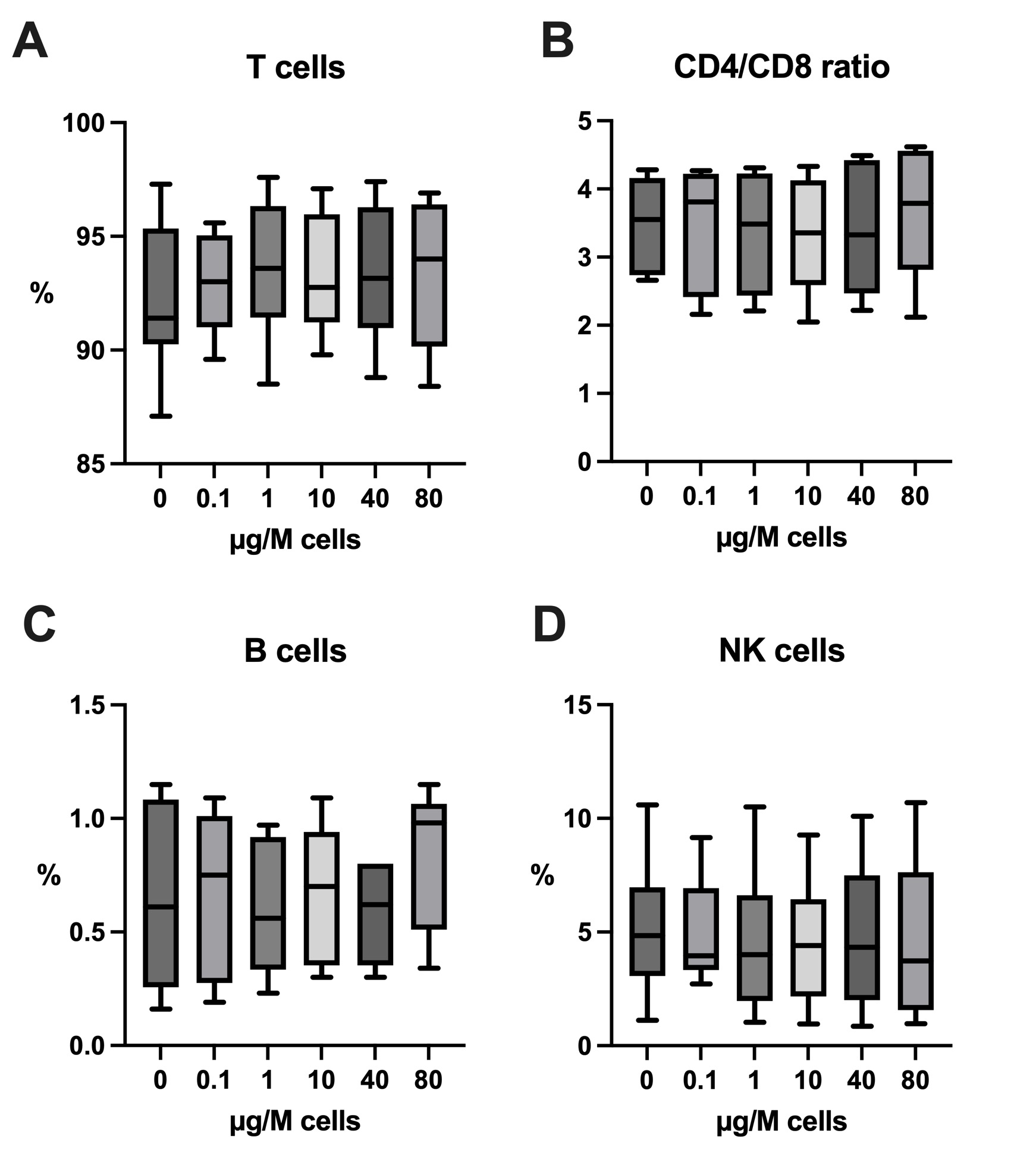

Supplement: S15 Fig — Comparison of DSIMC generation after two weeks of culture with increasing concentration of belatacept. T cells, NK cells, and B cells as percentage of total lymphocytes. No significant differences. FCM. (n = 6). (TIF) [file pone.0333356.s015.tif]

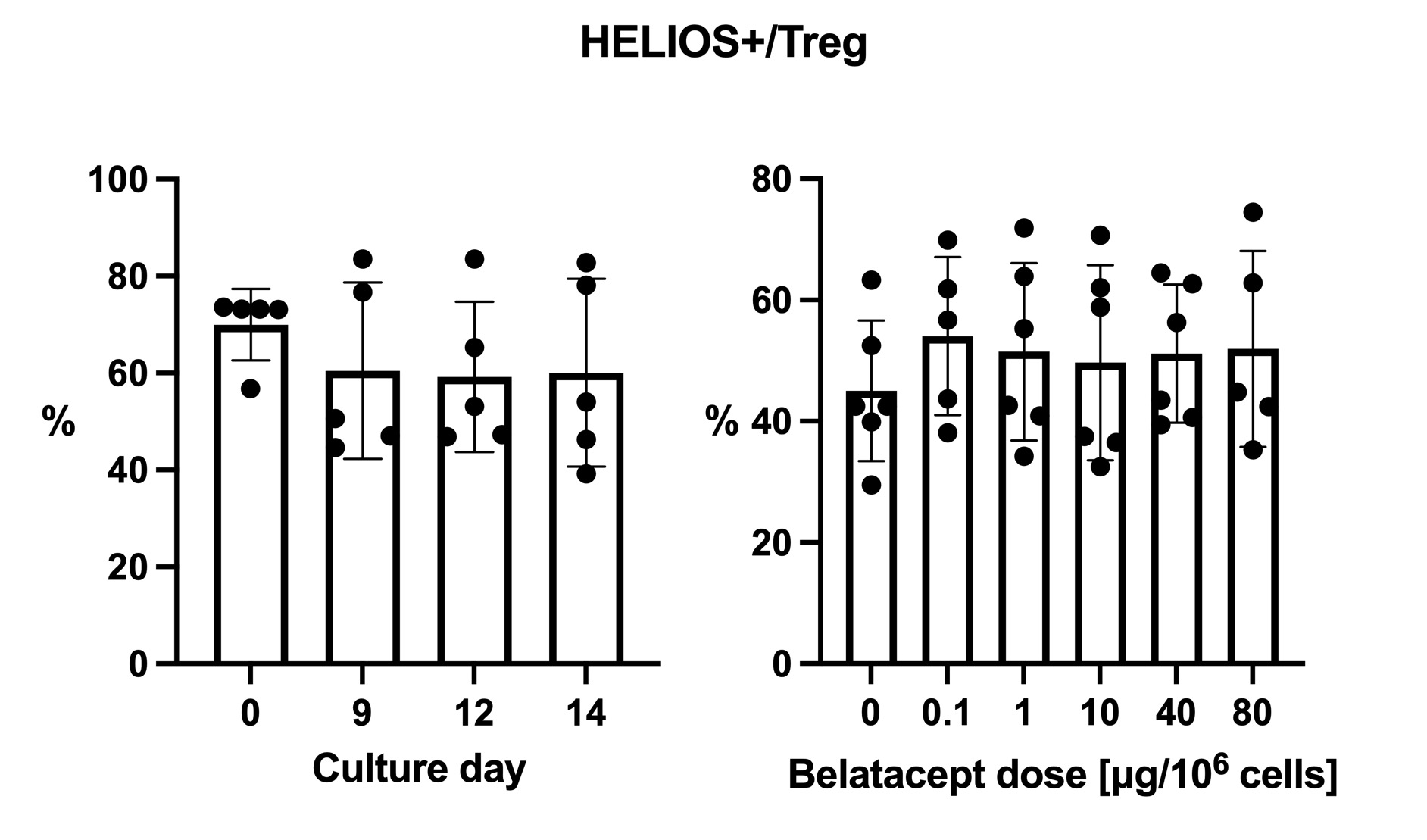

Supplement: S16 Fig — (Left) Comparison of HELIOS expression in Treg population over time. No significant differences. ANOVA. Means±SD. FCM. (n = 5). (Right) Comparison of HELIOS expression in Treg population of DSIMC after two weeks of culture with increasing concentration of belatacept. No significant differences. Mixed-effects analysis. FCM. (n = 6, two missing datapoints). (TIF) [file pone.0333356.s016.tif]

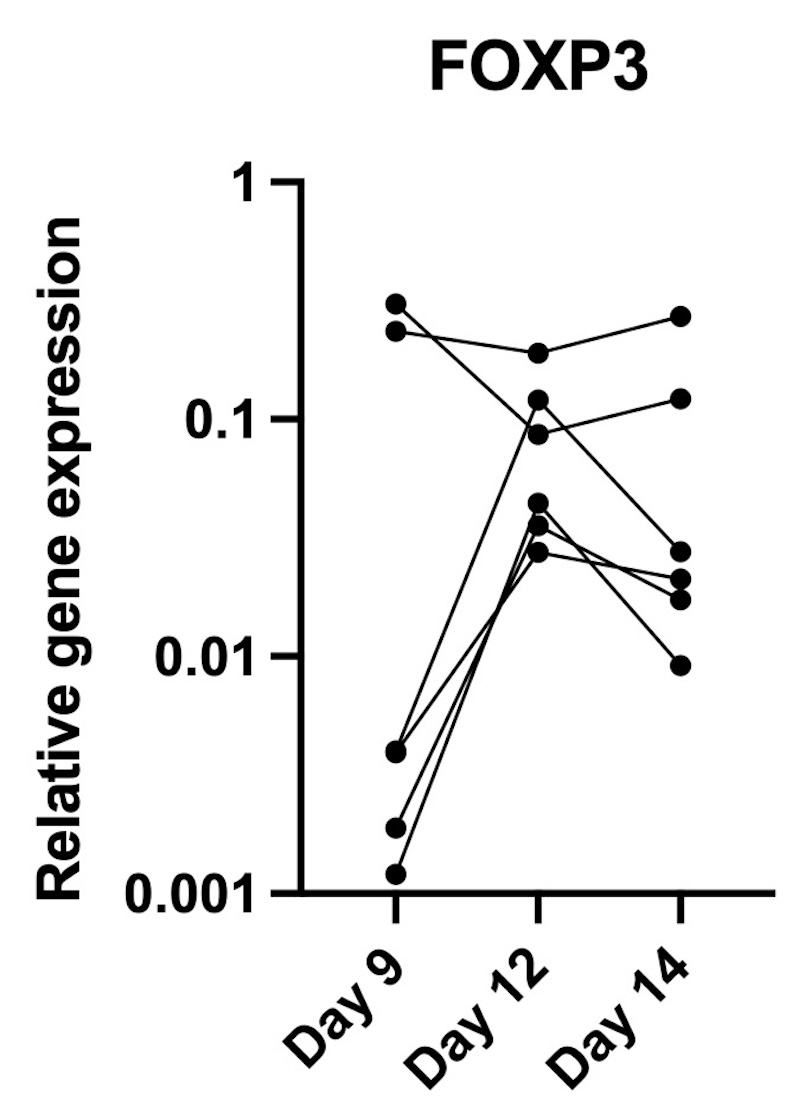

Supplement: S17 Fig — Comparison of FOXP3 expression in Treg population over second week of culture. No significant differences. One-way ANOVA. qPCR. (n = 6). (TIF) [file pone.0333356.s017.tif]

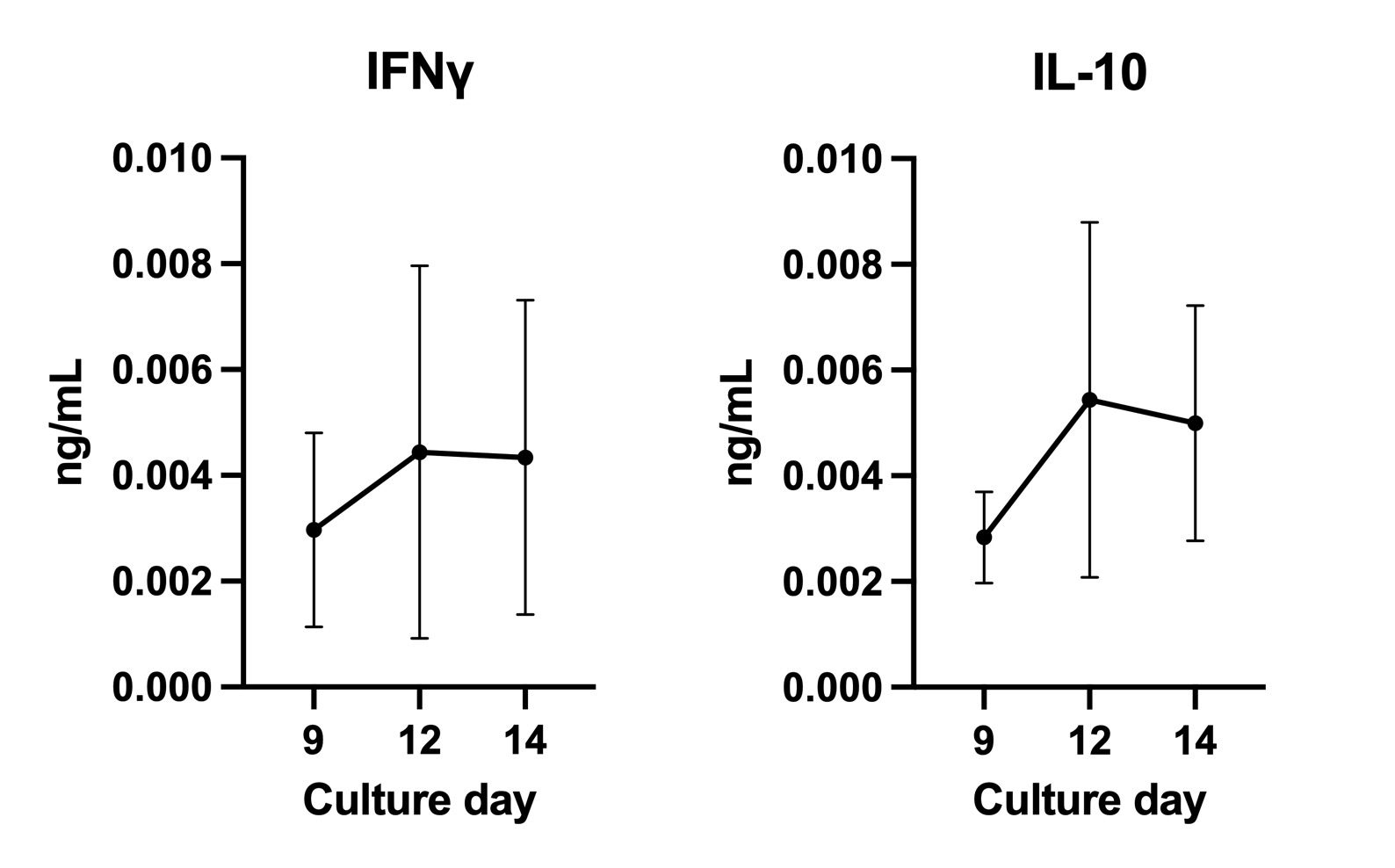

Supplement: S18 Fig — Comparison of IFNγ and IL-10 concentration in culture supernatant over second week of culture. No significant differences. One-way ANOVA. Means±SEM. ELISA. (n = 6). (TIF) [file pone.0333356.s018.tif]
