# Supplementary material for: Optimizing the in vitro production of immunomodulatory cells for the induction of tolerance in solid organ transplantation
Source: PLoS One. 2025 Nov 7;20(11):e0333356. doi: 10.1371/journal.pone.0333356 (PMC12594342; doi:10.1371/journal.pone.0333356)
Supplement: S1 Table — Treg (CD4 + CD25 + CD127lowFoxP3 + /CD45+) determined by FCM (n = 3). (DOCX) [file pone.0333356.s019.docx]

**Supporting table 1**. Cell yield, viability and %Treg of the generated cells in three different media. Treg (CD4+CD25+CD127lowFoxP3+/CD45+) determined by FCM (n=3).

|  | **Cell number x10^6^** | | **Viability %** | | **% Treg** | |
| --- | --- | --- | --- | --- | --- | --- |
|  | *Day 7* | *Day 14* | *Day 7* | *Day 14* | *Day 0* | *Day 14* |
| **Fresh cells** |  |  |  |  | 0.85±0.31 |  |
| **AlyS505** | 59.8±12.0 | 34.2±8.3 | 83.3±5.9 | 84.7±11.4 |  | 2.52±0.22 |
| **AIMV** | 45.7±2.6 | 33.0±7.5 | 82.7±6.8 | 89.6±8.8 |  | 2.70±0.45 |
| **TexMACS** | 46.6±2.7 | 28.1±1.7 | 80.4±5.8 | 78.9±6.2 |  | 3.28±0.28 |

Treg: regulatory T cells, FCM: flow cytometry.
